# From denial to awareness: a conceptual model for obtaining equity in healthcare

**DOI:** 10.1186/s12939-018-0723-2

**Published:** 2018-01-22

**Authors:** Anna T. Höglund, Marianne Carlsson, Inger K. Holmström, Linda Lännerström, Elenor Kaminsky

**Affiliations:** 1 Department of Public Health and Caring Sciences, Box 564, 751 22 Uppsala, Sweden; 2 University of Gävle, 801 76 Gävle, Sweden; 3 School of health, care and social welfare, Mälardalen University, Box 883, 721 23 Västerås, Sweden

**Keywords:** Equity in health, Conceptual model, Telephone nursing, Gender, Ethnicity, Sweden

## Abstract

**Background:**

Although Swedish legislation prescribes equity in healthcare, studies have reported inequalities, both in face-to-face encounters and in telephone nursing. Research has suggested that telephone nursing has the capability to increase equity in healthcare, as it is open to all and not limited by long distances. However, this requires an increased awareness of equity in healthcare among telephone nurses.

The aim of this study was to explore and describe perceptions of equity in healthcare among Swedish telephone nurses who had participated in an educational intervention on equity in health, including which of the power constructs *gender, ethnicity* and *age* they commented upon most frequently. Further, the aim was to develop a conceptual model for obtaining equity in healthcare, based on the results of the empirical investigation.

**Method:**

A qualitative method was used. Free text comments from questionnaires filled out by 133 telephone nurses before and after an educational intervention on equity in health, as well as individual interviews with five participants, were analyzed qualitatively. The number of comments related to inequity based on gender, ethnicity or age in the free text comments was counted descriptively.

**Results:**

Gender was the factor commented upon the least and ethnicity the most. Four concepts were found through the qualitative analysis: *Denial, Defense, Openness*, and *Awareness.* Some informants denied inequity in healthcare in general, and in telephone nursing in particular. Others acknowledged it, but argued that they had workplace routines that protected against it. There were also examples of an openness to the fact that inequity existed and a willingness to learn and prevent it, as well as an already high awareness of inequity in healthcare.

**Conclusion:**

A conceptual model was developed in which the four concepts were divided into two qualitatively different blocks, with *Denial* and *Defense* on one side of a continuum and *Openness* and *Awareness* on the other. In order to reach equity in healthcare, action is also needed, and that concept was therefore added to the model. The result can be used as a starting point when developing educational interventions for healthcare personnel.

## Background

Equity in healthcare has long been a guiding principle for healthcare policy in Sweden. A tax financed healthcare system and the Swedish Healthcare Act (1982:763), which prescribes equity in health for the whole population, have been regarded as guarantees for equity in healthcare. However, several studies have revealed discriminatory patterns in Swedish healthcare, related to factors such as gender, ethnicity and age. For example, it has been reported that women tend to be less likely to be prescribed expensive medicines and are less prioritized in healthcare than men [1]. Further, women with psoriasis were found to be offered self-care advice to a higher extent than men [2] and women often have to wait longer than men for ambulances and appointments with a general practitioner (GP) [1]. Similar gender related conditions have been reported internationally [3–5].

There are also gender differences in people’s care-seeking behaviour. For example, women tend to seek healthcare more actively than men do, and they are more eager to contact healthcare services on behalf of others, such as their children and partners [6, 7]. This tendency has also been observed internationally [8–11].

Hakimnia et al. [6] suggested that telephone nursing has the capability to increase equity in healthcare, as it is open to all and not limited by long distances from GP’s offices and hospitals. However, patterns of inequity have also been observed in this kind of care. For example, older age, often intersecting with gender, and foreign background with low language proficiency, have been shown to impact the use of National Health Service (NHS) Direct in the UK [12–15]. Also in Swedish telephone nursing, factors like gender, ethnicity and low proficiency in Swedish, as well as high age, have been identified as influencing the use of the service [6, 7, 16].

Telephone nursing is an expanding service in many countries, e.g. the UK, Australia and the US [17]. Sweden is in the front-line, with all counties connected to Swedish Healthcare Direct (SHD), with a common telephone number, 1177. The intention of the service is to make healthcare more efficient, but also more accessible and safe for patients (www.1177.se).

A telephone nurse’s task is to assess symptoms and direct the caller to the right level of healthcare, or to give self-care advice. Telephone nurses in Sweden handle a least six to eight calls per hour [18]. They are obliged to use a computerized decision support tool (DST). Assessing, referring and giving advice, but also being supportive and/or teaching the caller, are important aspects of telephone nursing [18].

For the past 20 years, Swedish healthcare has undergone key changes, with New Public Management (NPM) reforms and privatization at its center [19, 20]. The idea behind NPM is to transfer ideals and methods from the private to the public sphere. Still, no more than 10% of the Swedish care providers are private and few Swedes have private health care insurance (svenskforsakring.se/en). Telephone nursing can be seen as an example of healthcare produced according to NPM. It is a service inspired by call centers at private companies [21] and has been seen as a cost-effective way to provide healthcare [22]. As previous research has reported that use and advice in this system is related to factors such as gender, ethnicity and age [6, 7, 13–15], it seems imperative for telephone nurses to improve their awareness of what equity in health means and how it can be implemented. Further, Kaminsky et al. [23] have identified the lack of theoretical research approaches within this field, and the present article is an attempt to fill in this gap.

### Theoretical framework

Justice and equity are concepts that deal with moral judgements of differences between people and their life conditions. A well-established understanding of equity is that, based on the principle of human dignity, all persons have the same human rights and are entitled to have these rights respected [24]. From that it can be concluded that the goods of society should be distributed equally. In its basic interpretation, this would mean that all persons should be given the same amount of the good that should be distributed in a society, but as that is rarely the case, several theories within political philosophy and social ethics have focused on the moral justifications for exceptions from this fundamental rule [25].

Justice can be seen both as a distributive concept (focused upon how the goods of a society should be divided) and as a retributive concept (focusing on just treatment or punishment according to deeds). In healthcare, mainly justice as distribution is relevant, as it concerns the distribution of social goods in a society. There are different understandings of what constitutes a just distribution of social goods. John Rawls [26] argued that the societal distribution of goods should be equal, unless an unequal distribution was in the favor of the least privileged groups in society. Further, Rawls argued that *social goods*, in the form of, for example, healthcare, education and social services, should be used to compensate for inequalities caused by the unequal distribution of *natural goods*, such as endowments and inherent health conditions.

According to Michael Walzer [27], s*imple equality* means that everyone should have exactly the same amount of social goods, for example, money. *Complex equality*, on the other hand, means that an unequal distribution could be accepted within one specific sphere of society, as long as it does not automatically give advantages in other spheres. For example, the possession of one type of social good, such as money, should not automatically give access to other social goods, such as healthcare. Walzer refers to this as “spheres of justice”.

Justice, equity and equality, are also important concepts in gender research. According to well-established definitions, *gender equality* concerns the absence of discrimination between women and men, whereas *gender equity* is about men’s and women’s needs, e.g., health needs, whether similar or not [28]. In the present study, gender equity is primarily relevant, as it concerns the distribution of healthcare resources. However, the concepts are connected, as lack of gender equality, i.e., discrimination due to gender, can prevent healthcare staff from promoting gender equity, i.e., providing healthcare according to gender-based needs, because of prejudices and stereotyped understandings of masculinity and femininity.

Current gender research often includes theories on intersectionality [29–31]. According to this theoretical position, gender interacts – or intersects – with other categories, such as ethnicity and age. According to Lykke [32, 33], theories on intersectionality aim to analyze how sociocultural hierarchies and power interact and construct inclusion or exclusion according to factors such as gender, ethnicity, age, sexuality and dis/ability. The focus is on the interaction between different constructs of power and the consequences this can have for individuals and societies.

We argue, that one strength of intersectionality theories is that they acknowledge the complexity in how different social power constructs intersect. If analyzed through the lens of Walzer’s theory on spheres of justice and complex equality [27], the intersection of different categories risks that one type of social favors, for example gender, intersects with ethnicity or language proficiency, and thereby gives access to other favors, such as high priority in healthcare, independently of healthcare needs.

As mentioned above, studies have reported inequalities in healthcare providing in Sweden, both in face-to-face encounters [1, 2] and in telephone nursing [6, 7, 16]. Given the political goal of equity in health in Sweden, an increased awareness of this issue is crucial for healthcare providers in general and, we argue, for telephone nurses in particular. They are often the primary providers of care and work through a technology that has the potential to make healthcare more available for persons having difficulties to achieve face-to-face encounters, due to factors such as age, language or regional distance.

### Aim

Against this background, the aim of the present study was to explore and describe perceptions of equity in healthcare among Swedish telephone nurses who had participated in an educational intervention on equity in healthcare, including which of the power constructs *gender, ethnicity* and *age* they were most likely to comment upon. Further, the aim was to develop a conceptual model for obtaining equity in healthcare, based on the results of the empirical investigation.

## Method

The design was explorative and qualitative. The present study is part of a larger project on equity in telephone nursing in Sweden. Within that project, a study-specific questionnaire was developed. Results from that instrument have previously been reported [34]. The questionnaire was also used in an intervention study, in which two telephone nursing sites in Sweden were included in a quasi-experimental study, where one site received an educational intervention on equity in health, while the other functioned as a control group [35]. In the present study, results from the intervention study which have not previously been reported are presented, based on the answers to free text comments in the study-specific questionnaire. Further, results from individual interviews with five participants from the intervention group are presented.

### Participants

Telephone nurses from two different sites in Sweden were asked to participate in the study. The sites were strategically chosen, since they were similar in size and in number of employed telephone nurses (30–40 persons). One site functioned as the intervention group and the other as the control group. Mean age in the intervention group was 57, both before and after intervention. In the control group, the mean age was 58 before intervention and 56 after intervention. All but two participants were women, and therefore differences in answers between male and female participants were not reported, as that risked violating the respect of confidentiality. Characteristics of the participating telephone nurses are presented in Table 1.

**Table 1:** Characteristics of participants

	Intervention group				Control group			
	Before intervention (*n* = 32)		After intervention (*n* = 25)		Before intervention (*n* = 41)		After intervention (*n* = 35)	
Age	Mean: 57.28	Range: 39–72	Mean: 57.21	Range: 34–72	Mean: 58.39	Range: 42–71	Mean: 56.09	Range: 28–67
Fulltime work	14 (43,8%)		6 (24,0%)		12 (29,3%)		10 (28,6%)	
Part time work	17 (53,1%)		19 (76,0%)		27 (65,8%)		23 (65,7%)	
Employed on hourly basis	1 (3,1%)		0 (0%)		2 (4,9%)		2 (5,7%)	

All in all, 73 telephone nurses participated in the baseline measurement (32 in the intervention group and 41 in the control group). After the intervention, described elsewhere [35], in the follow-up evaluation, 60 telephone nurses participated (25 in the intervention group and 35 in the control group). Drop-outs were due to factors such as sick leave and change of work place. Hence, all in all, 133 answered questionnaires formed one part of the material for the present study. Apart from that, individual interviews were made with five telephone nurses, all women, who had participated in the educational intervention.

### Procedure

Permission to perform the study was given by the respective head of the sites. All telephone nurses working on the sites were asked to participate in the intervention study and they were included after individual informed consent. The questionnaire was developed by the research group and is described elsewhere [34, 35]. It measures three aspects of power, namely, gender, age and ethnicity, and includes descriptions of twelve hypothetical persons, of different age and gender, born in Sweden or outside Europe. The hypothetical persons are described in Table 2.

**Table 2:** Description of the fictive persons in the study specific questionnaire and number of comments on the fictive persons

Name	Age	Gender	Swedish or Non-European	Number of comments in the 133 questionnaires
Isa	25	Female	Swedish	16
Lynn	25	Female	Non-European	21
Johanna	45	Female	Swedish	14
Manuela	45	Female	Non-European	19
Karin	70	Female	Swedish	14
Li-Xing	70	Female	Non-European	21
Alexander	25	Male	Swedish	15
Elliot	25	Male	Non-European	20
Björn	45	Male	Swedish	8
Urgesa	45	Male	Non-European	23
David	70	Male	Swedish	17
Ahmed	70	Male	Non-European	18

The items in the questionnaire concerned assessments of the likelihood of whether a hypothetical person had called SHD, whether or not s/he was recommended to have an appointment with a doctor when calling, whether s/he had a high quality of life, power over his/her life and whether s/he had experienced discrimination. Each participant was asked to assess two of the hypothetical persons, randomly selected [34, 35]. The questionnaire items are described in Table 3. The participants were also asked to add comments to their assessments, using free text, and the present article reports the results from these free text comments.

**Table 3:** Description of the questionnaire

Description of two hypothetical persons, e.g. Isa, 25 years old and born in Sweden, and Urgesa, 45 years old and born outside Europe. For each person the following questions are asked:
How likely is it that s/he:
1. Has called SHD 1177?
2. Is recommended a doctor’s appointment when calling 1177?
3. Has high quality of life?
4. Has power over his/her own life?
5. Is living alone?
6. Has addiction problems?
7. Is working?
8. Has experienced discrimination?
9. Has been on long sick leave?
10. Have you answered in the same way for the two hypothetical persons? (Yes or no)
11. If no: On what question/s did your answers differ for the two hypothetical persons?
12. Please, try to give a short explanation to why your answers differed.
13. Free text comments.

The baseline questionnaire was distributed to the intervention group and the control group during workplace meetings in October and November 2014. The follow-up questionnaire was distributed to both groups on workplace meetings in May and June 2015, i.e., approximately six months after the intervention.

In order to further evaluate the intervention, all participants in the educational intervention (*n* = 19) were asked to participate in an individual interview. Five telephone nurses, all women, who accepted the invitation to be interviewed were included after informed consent. The interviews were made over the phone in September 2015 and performed by one of the authors (LL). An interview guide was developed, covering questions of how the interviewees had experienced the educational intervention, how they defined equity in health and whether they regarded it as an important aspect of healthcare. Further, the guide asked for suggestions of how awareness of equity in healthcare could be developed and upheld in the telephone nurses’ daily work. The complete interview guide is described in Table 4.

**Table 4:** Description of the interview guide

1. What are your perceptions of participating in the course on equity in healthcare?
2. What does “equity in healthcare” mean to you?
3. Do you perceive equity in healthcare to be an important issue in healthcare providing?
4. What other questions could be important in healthcare providing?
5. How has your daily work been impacted by the educational intervention?
6. Can you give examples of discussions you’ve had on your workplace, as a result of the intervention?
7. Apart from educational courses, how do you think awareness of equity in healthcare could be improved?
8. Do you have anything to add, in relation to the educational intervention on equity in health?

### Analysis

The free text comments in the questionnaire were gathered verbatim in a separate document. The individual interviews were recorded and transcribed verbatim. The material was analysed by content analysis as described by Elo & Kyngäs [36]. All transcripts were read through thoroughly and meaning units were identified and grouped together and sorted into concepts. According to Elo and Kyngäs [36] the aim of a content analysis is to attain “a condensed and broad description of the phenomenon, and the outcome of the analysis is concepts or categories describing the phenomenon” [36], p. 108. Further, Elo & Kyngäs argue that the purpose of such concepts is to build up “a model, a conceptual system, a conceptual map or categories” (ibid.). This was also the case in this study, as one aim was to develop a conceptual model for obtaining equity in healthcare.

We also counted how many times comments on the different hypothetical persons were made, as well as how many times the studied aspects (gender, ethnicity and age) were commented upon, in relation to how many times it was possible to comment upon them.

The initial analyses of the material were made by two of the authors (ATH and EK). Thereafter, all authors discussed and revised the suggested analysis and categorization. When disagreements occurred, discussions took place until consensus was reached. The final version was consented to by all authors.

### Ethical considerations

Approval for the study was sought at the Regional Ethics Review Board (Dnr 2014/130), but the Board found that according to the Swedish Act Concerning the Ethical Review of Research Involving Humans (SFS 2003:460), no formal approval of the project was needed, as it did not deal with sensitive personal data or risk impact on the participants physically or psychologically. Throughout the project, however, the ethics of scientific work as outlined in the Declaration of Helsinki [37] were followed. Permission to perform the study was given by the respective head of the sites. The telephone nurses were informed both orally and in writing about the study and were included after informed consent. Information emphasized that participation was voluntary and possible to withdraw from at any time. Likewise, it was stressed that data would be handled confidentially and that no workplace or individual would be identifiable in the reporting of results.

## Results

There were no notable differences between the intervention group and the control group concerning the free text comments. However, when counting how many times comments were made on the different power aspects that the questionnaire covered (gender, ethnicity and age), some patterns emerged. First, it was found that gender was the factor that was commented upon the least in the material. Such comments were made in 56% of the possible cases (30 out of 54). Ethnicity, on the other hand, was the most frequently commented upon factor as such comments were made in 81% of the possible cases (72 out of 89). Also, age was more frequently commented upon than gender. When there were possibilities for comments about age, this was made in 77.5% of the answers (55 out of 71 possible times).

Second, the analysis revealed that the hypothetical persons with foreign sounding names and with non-European background were more frequently commented upon than the hypothetical persons who were born in Sweden and with Swedish-sounding names. The non-European hypothetical persons were commented upon 122 times in the 133 analyzed questionnaires, whereas the hypothetical persons with Swedish names were commented upon 84 times (Table 2).

Finally, we found that when comparing two hypothetical persons with the same age (45 years old) and the same gender (male), but with different background, in this case Björn, who was said to be born in Sweden and Urgesa, who was said to be born outside Europe, the comments on the non-Swedish person were almost three times higher, as Björn was commented upon 8 times while Urgesa was commented upon 23 times in the 133 analyzed questionnaires (Table 2).

Through the content analysis of both interviews and free text comments in the questionnaire four main concepts were developed, namely *Denial; Defense; Openness,* and *Awareness*. In the following, each concept is presented by quotes.

### Denial

The first concept, “Denial”, captures the fact that some informants denied the existence of inequity in healthcare in general, and in telephone nursing in particular, and also, to some extent, in society as a whole. Several informants mentioned that healthcare was provided equally, according to them, and that inequity in health was not a problem, at least not in telephone nursing.

*It was not such big news. I have read research reports before about men being prioritized… But I don’t recognize that from my own work*. (Interview #4)

*I make judgements solely based on symptoms for each person at every occasion*. (Free text comment, woman, intervention group after intervention)

*A patient is a patient, independent of gender, age or nationality.* (Free text comment, woman, intervention group before intervention)

In line with this, there were also answers that expressed frustration over the questionnaire, which was described as being based on prejudices. This was, however, only found in the free text comments in the questionnaire and was not mentioned in the individual interviews.

*It feels as if those who have developed the questionnaire really want to emphasize inequalities in the encounter [between patient and healthcare provider].* (Free text comment, woman, intervention group after intervention)

*It’s impossible to answer these questions, because of the lack of information*. (Free text comment, woman, intervention group after intervention)

*Unanswerable questions! It’s all just guessing!* (Free text comment, woman, intervention group after intervention)

### Defense

The concept “Defense” contains examples of how the informants to some extent acknowledged the existence of inequity in healthcare, but still put forward that they worked against this. Further, they expressed that there were working routines that protected from inequities in the telephone nurses’ providing of healthcare advice. Referrals were primarily made to the computerized DST Swedish telephone nurses are obliged to use, which was assumed to prevent unequal treatment.

*I assess needs according to symptoms for each person at every occasion.* (Free text comment, woman, intervention group after intervention)

*To recommend a doctor’s appointment is always based on how severe the illness is.* (Free text comment, woman, control group before intervention)

*Independently of work position, education, or skin color, you should be entitled equal care and equal opportunities. There should be no differences whatsoever, concerning what care you are offered*. (Interview #2)

*Irrespectively of who is calling, you should be as neutral and professional as possible, so that you are not impacted by the person who is calling.* (Interview #3)

### Openness

However, not all informants were denying the existence of inequity in healthcare, nor defending themselves from being part of it. The concept “Openness” concerns the fact that several informants expressed a readiness to learn more on equity in health and develop their competence in these questions. One frequently mentioned opinion was that equity in health is an important question for the healthcare system.

*I think these kind of questions are so interesting, because… well, it’s weird, but the encounter can differ based on who you are*. (Interview #2)

*It’s an interesting question, because you think that you are treating everyone as equals, or you hope you do that /---/ and then we were shown that, maybe unconsciously, we do prioritize in a certain way. I thought that was a really interesting subject to discuss!* (Interview #4)

Examples of safety-netting were also found within this category, in that some telephone nurses expressed that they might prioritize appointments to a GP for persons with low proficiency in Swedish, in case they had missed some important information in the call because of the low language proficiency of the caller. Here, no explicit awareness of inequity was expressed, but the measures taken in order to prevent unequal treatment can be regarded as being open to the fact that discrimination can occur.

*Because of language difficulties, callers with foreign background are often given priority for a doctor’s appointment.* (Free text comment, woman, intervention group after intervention)

*We often send someone who don’t understand the language so well to a GP, just to be sure we aren’t missing anything.* (Free text comment, woman, intervention group before intervention)

### Awareness

The final concept, “Awareness”, captures answers that express awareness among the informants of inequity in healthcare. Further, it contains examples of strategies that the informants used to promote equity in their giving of healthcare advice, i.e., strategies they used in order to prevent the discriminatory structures they were aware of.

Many power constructs that could cause experiences of discrimination for callers were mentioned within this category, for example, ethnicity and age.

*The Swedish society is very discriminatory, especially against people born abroad, who often have difficulties in finding a job.* (Free text comment, woman, intervention group before intervention)

*The elderly, who might have difficulties to call, they might experience this new technology as difficult, they don’t understand the system. And newly arrived immigrants, who don’t know the language. (Interview #4)*

*Well, you try not to treat patients differently, but something happens, depending on who is talking. But I don’t think it’s only about where you come from, if you’re foreign, but it depends on… well, if you can speak up for yourself. (Interview #2)*

Examples of intersection between different power constructs were also expressed, for example ethnicity and gender.

*A woman who is an immigrant is worse off, compared to a man who is immigrant*. (Free text comment, woman, control group before intervention)

Comments on the inequality between women and men in Swedish society were also quite common.

*A man takes up more space in society, whether he is Swedish or not. (−−-) Men have stronger positions than women, women have to stand back.* (Free text comment, woman, intervention group before intervention)

Further, sexual orientation was mentioned as an aspect that could lead to discrimination.

*Those who don’t live in stereotypical nuclear families. That can also be something to think of. The last 10 or 20 years, a lot has happened concerning that matter.* (Interview #5)

In addition, the intersection of different factors was mentioned, for example, gender and socio-economic status were identified as aspects that could create inequalities between women and men. Male gender and high socio-economic status could result in better care, according to some informants.

*Men with high education and good jobs, who know what they want and what they are capable of getting, get better care, simply enough. Higher care level than motivated, so to say. Because you don’t bother to argue with them… You can often be treated badly if you do, unfortunately.* (Interview #2)

Apart from inequalities due to gender, ethnicity and socio-economy, other forms of inequality were also mentioned, for example, between different regions in Sweden.

*We in this county don’t have the same opportunities as they have in Stockholm. We don’t! So, it can be very different throughout the country, what tools we have to work with*. (Interview #1)

The participants also suggested strategies for achieving greater awareness of equity in healthcare. Primarily, the educational intervention was mentioned as a good example of how awareness could be developed.

*During the education session, all of us admitted that we have prejudices. It’s a big step, just to admit that!* (Interview #1)

*I would like a longer education session, to go deeper into these questions. /---/ More and longer courses, several times a year, perhaps*. (Interview #2)

The informants further described how they, due to their awareness of inequalities in society, tried to identify members of vulnerable groups, so that they could give special attention to them.

*Women with foreign background who don’t speak Swedish, they might be excluded, so then I try myself to give them what they need*. (Interview #2)

**Fig. 1:**
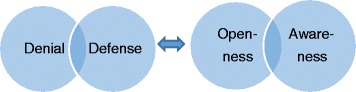
Description of the four concepts as two qualitatively different blocks

The four concepts, *Denial, Defense, Openness* and *Awareness*, can be seen as positions on a continuum, where an individual, or a group of individuals, can move from one position to another. One way to interpret such a continuum is that an individual can develop and mature and thereby go from *Denial*, via *Defense* and *Openness*, to *Awareness*. If seen as such a continuum, awareness becomes the final position, which is then the most desirable one. However, it is also possible to argue that the same individual can shift between different positions on the continuum, depending on context and circumstances. This is illustrated in Fig. 1.

To the left of the figure *Denial* and *Defense* are found, as they are constitutively alike in refraining from the admission of inequity in healthcare. To the right, we find the concepts *Openness* and *Awareness*, as these are united in the acceptance of inequity in healthcare and an ambition to change this and obtain equity in healthcare. This will be further elaborated in the Discussion section below.

## Discussion

One aim of the present investigation was to explore and describe perceptions of equity in healthcare among Swedish telephone nurses who had participated in an educational intervention on equity in health. A motive for directing such an educational intervention to telephone nurses is that they are often the primary providers of care. Further, they work with a technology that has the potential to make healthcare more equal, which has previously been argued for by Hakimnia et al. [6]. Thereby, telephone nursing can improve healthcare contacts for persons who have difficulties achieving face-to-face encounters with healthcare providers, due to factors such as age, language or regional distance.

The analysis showed that gender was the factor commented upon the least and ethnicity the most. These results support previous findings, showing a resistance towards work for equality between women and men, based on the assumption that such equality has already been reached in the Nordic countries [38]. They are also in line with the results from our previous studies, using the same questionnaire [34, 35].

The qualitative content analysis, based on individual interviews and free text comments in the study-specific questionnaire, resulted in four concepts, namely *Denial, Defense, Openness* and *Awareness*.

*Denial* concerned the fact that some informants denied the existence of inequity in healthcare in general, and in telephone nursing in particular. *Defense* described how the informants to some extent acknowledged the existence of inequity in healthcare, but still put forward that they worked against this and also that there were working routines that protected from inequity in the telephone nurses’ provision of healthcare advice. Particularly, the computerized DST, which Sweden telephone nurses are obliged to use, was mentioned as such a routine. Previous research has revealed that Swedish telephone nurses apprehend DST as both supportive and obstructive in their work [39], but in our study the system was primarily described as helpful.

Our results concerning *Denial* and *Defense* are in line with the study by Hølge-Hazelton & Malterud [38], who found that a notion of gender neutrality is still alive in the medical context, although not supported by empirical evidence. In accordance with this, some informants claimed that they encountered every patient as an equal human being, without prejudices, although research has revealed inequity in Swedish healthcare, related to factors such as gender and ethnicity [1].

*Openness* captured that some informants expressed a readiness and willingness to learn more about equity in health and develop their competence in these questions. *Awareness*, finally, showed that several informants were aware of inequalities in healthcare – and in society as a whole - and also that they were eager to work against inequity through active strategies, for example, by paying certain attention to what they had identified as vulnerable groups.

This finding can be understood in light of Rawls’ theory of justice [26], according to which an equal distribution of societal goods is to be aspired for, unless an unequal distribution is in favor of the least privileged groups in the society.

We also found that the informants’ awareness concerned not only the existence of one form of discrimination, but rather the intersection of different categories [32, 33]. For example, gender and ethnicity were put forward as examples of factors that could interact with and increase the experience of discrimination for certain individuals. Additionally gender in connection with socio-economy was described as a form of intersection between categories that could create inequality. This is in line with our previous studies, in which we found that both Swedish nursing students and telephone nurses in Sweden had quite a high awareness of how power and gender, ethnicity and age, are related [34, 35].

Our results can also be seen through the lens of complex equality, as developed by Walzer [27]. Sociocultural hierarchies and power can intersect and construct inclusion or exclusion in society’s welfare system. According to Walzer’s reasoning, this should be opposed, as it is an example of how advantages within one societal sphere also give advantages within other societal spheres, which is against the principle of justice [27].

The informants also gave examples of strategies for improving awareness of equity in health. Education, preferably in the form of longer courses several times a year, as well as opportunities for discussions on these issues were mentioned. The educational intervention described in a previous article [35] is an example of such an effort, providing time for discussions.

### The conceptual model

One aim of the present study was to develop a conceptual model for obtaining equity in healthcare, based on the results of the empirical investigation. We argue, that the concepts found in the qualitative analysis can form the basis for a conceptual model of the phenomenon equity in healthcare.

As mentioned above, the four concepts (*Denial, Defense, Openness* and *Awareness*) can be seen as positions on a continuum. It is also possible to regard them, not as four separate positions of a continuum, but as *two qualitatively different blocks*, where *Denial* and *Defense* are on one side of the model, and *Openness* and *Awareness* are on the other side. The strength of this model is that it acknowledges how *Denial* and *Defense* are connected by the resistance to acknowledging existing discriminatory structures and, hence, both positions describe a form of opposition towards increasing awareness of inequalities and active actions for equity. Likewise, the model makes visual how the concepts *Openness* and *Awareness* are connected, in that they stand for accepting the imperfect situation in healthcare today and striving for change of discriminatory structures (see Fig. 1).

Based on the results of our study, we suggest a combination of the two models described above. On the one hand, we argue that the categories, *Denial* and *Defense,* are qualitatively different in character from *Openness* and *Awareness* and therefore could be seen as two separate blocks. On the other hand, there is an advantage in regarding the four categories as a continuum, as it makes room for improvement and development, both within individuals and in workplaces. We therefore argue for a combined model, which acknowledges both the resemblance and overlap between *Denial* and *Defense* on the one hand and between *Openness* and *Awareness* on the other hand; as well as interprets the different categories as positions on a continuum.

**Fig. 2:**
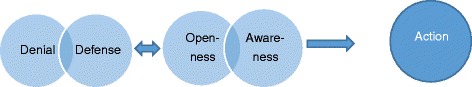
Description of the conceptual model with the four concepts as two qualitatively different blocks, but also as positions on a continuum, and with *Action* as a fifth concept

However, in order to reach equity in healthcare, action is also needed. This has been discussed in relation to improving ethical awareness in healthcare by, e.g., Eriksson et al. [40] and Höglund et al. [41], who argued that such awareness includes the ability to perceive ethically challenging situations, judge them and act upon them. Arguably, awareness of in/equity in healthcare can be interpreted similarly. Therefore, the concept of *Action* needs to be added to the model, although it was not found as a separate concept in the analysis. It could, though, be understood as implied in the concept of *Awareness*, as the quotes within this concept described examples of actions, such as identifying vulnerable groups and paying special attention to them. We choose to make *Action* visible as a certain concept, as shown in Fig. 2.

If seen in this way, it is possible to argue that although *Denial* and *Defense* are qualitatively different from *Openness* and *Awareness*, they can also be seen as a continuum, making it possible to move from *Denial* to *Awareness* through, for example, education and courses. The ideal movement is from left to right, i.e., from *Denial* to *Action*, but a realistic scenario might be that the same individual moves back and forth on the continuum. Therefore, regular training and discussions, as suggested by the informants in our study, are of the utmost importance in the work for equity in healthcare.

### Strengths and limitations

A strength in the present study was the rich material the analysis was built upon. The data consisted of both individual interviews and answers to free text comments in a questionnaire. The research group is multi-disciplinary, consisting of expertise in nursing (EK, IKH, LL), ethics (ATH), and psychology (MC).

Almost all informants were women, which can be seen as a weakness. Further, the mean age was quite high among the informants (56–58 years) and almost all participants were of Swedish origin. However, this mirrors the situation within the workforce of telephone nurses in Sweden. Some participants were over 67 years old, which is the retirement age in Sweden. Due to the current shortage of nurses in Sweden, retired nurses are sometimes hired by SHD sites for shorter periods of time, which can explain the high mean age of the participants in the study.

Despite repeated contacts with the telephone nursing sites, we did not manage to include more than five telephone nurses for the individual interviews within the time limits of the project. This could be due to the telephone nurses’ already heavy work schedule, but also to the fact that they had recently participated in the educational intervention and did not want to discuss these questions again after such a short period of time. In spite of the few participants, the results of the interviews were well in line with the free text comments of the questionnaire, which makes the results credible.

The study was made in Sweden, but we argue that the results are transferable to other contexts with similar systems, such as NHS Direct in the UK.

## Conclusion

The studied telephone nurses expressed both denial and defense in relation to work for equity in healthcare. At the same time, examples were found of both openness to learning more about these issues, as well as an already quite high awareness. The participants were most inclined to comment on ethnicity as a factor of discrimination, while gender was the category that was commented upon the least. A conceptual model, based on the empirical results, were developed, showing how *Denial* and *Defense,* on the one hand are related, as well as *Openness* and *Awareness* on the other. However, awareness is just the first step to change; active actions must also be taken in order to reach increased equity in healthcare.

The results can be used as a starting point when developing new educational interventions on equity in healthcare. Such interventions can benefit from being adapted to the group that is being taught. One way to do this is to start with a survey of the group, to get to know where on the suggested continuum the participants are situated, in order to customize the educational course for the particular group.

## Abbreviations

DST: Decision Support Tool
GP: General Practitioner
NHS: National Health Service
NPM: New Public Management
SHD: Swedish Healthcare Direct
